# Clinical characteristics and risk factors for poor outcomes of invasive pneumococcal disease in pediatric patients in China

**DOI:** 10.1186/s12879-024-09493-9

**Published:** 2024-06-19

**Authors:** Yanan Fu, Yingchun Wang, Wei Tang, Qing Yang, Guan Wang, Meng Li

**Affiliations:** 1 Department of Medical Engineering, Qilu Hospital of Shandong University, Jinan, Shandong Province China; 2 Department of Pediatrics, Qilu Hospital of Shandong University, No.107 West Wenhua Road, Jinan, 250012 Shandong Province China

**Keywords:** Children, Antimicrobial resistance, Mortality, IPD, Sepsis

## Abstract

**Background:**

Invasive pneumococcal disease (IPD) is a significant health concern in children worldwide. In this study, we aimed to analyze the clinical features, antibiotic resistance, and risk variables for poor outcomes in patients with IPD in Hangzhou.

**Methods:**

A retrospective single-centre study was performed using the pediatric intensive care (PIC) database from 2010 to 2018. The clinical characteristics, laboratory data, antimicrobial resistance, and risk factors for in-hospital mortality and sepsis in patients with IPD in intensive care units (ICUs) were analyzed systematically.

**Results:**

A total of 178 IPD patients were included in the study. The majority of the IPD children were 2–10 years old. Antimicrobial resistance tests of *S. pneumoniae* isolates revealed high resistance to erythromycin, tetracycline and compound sulfamethoxazole (SMZ-Co). All the isolates were sensitive to vancomycin, linezolid, moxifloxacin, telithromycin, ofloxacin, and levofloxacin. IPD patients may experience poor outcomes, including death and sepsis. The in-hospital mortality was 3.93%, and 34.27% of patients suffered from sepsis. Temperature (OR 3.80, 95% CI 1.62–8.87; *P* = 0.0021), Partial Pressure of Oxygen in Arterial Blood (PaO_2_) (OR 0.99, 95% CI 0.98-1.00; *P* = 0.0266), and albumin (OR 0.89, 95% CI 0.80–0.99; *P* = 0.0329) were found to be independent risk factors for sepsis in children with IPD.

**Conclusion:**

Pediatric IPD deserves attention in China. Appropriate surveillance and antibiotic selection are crucial in managing resistant strains. Early identification of high-risk individuals with risk factors contributes to the development of appropriate treatment strategies.

**Supplementary Information:**

The online version contains supplementary material available at 10.1186/s12879-024-09493-9.

## Background

*Streptococcus pneumoniae* (*S. pneumoniae*) is a leading cause of serious infectious diseases associated with significant morbidity and mortality worldwide [1]. *S. pneumoniae* typically colonises the pharynx and the upper respiratory tract of healthy individuals. However, when the host immune function is weak, the pathogen can cause invasive pneumococcal disease(IPD) which can be transmitted to various sterile sites, such as the blood, cerebrospinal fluid (CSF), pleural space, and peritoneal fluid [2]. The mortality rate of children with IPD is approximately 5.3-27.5% [3–5], with more than 1 million deaths recorded annually worldwide; moreover, approximately 25-50% of survivors develop serious neurological sequelae [6, 7]. A surveillance report showed that China had the largest number of patients with pneumococcal-associated diseases compared with 12% among ten African and Asian countries; furthermore, approximately 30,000 children younger than five years of age died from IPD in 2000 [7]. With the widespread application of pneumococcal conjugate vaccines (PCVs), the incidence of IPD significantly declined by 51% between 2000 and 2015 [8]. However, PCV vaccination is not routinely considered by governments of many countries, including China, and the immunisation rate is generally low. Consequently, the incidence and mortality rates of the disease are high in these countries.

The clinical signs and symptoms of IPD vary among different cases and are related to host immunity, bacterial virulence, and the infection site [9]. Moreover, the incidence of antibiotic resistance in *S. pneumoniae* has increased in recent years owing to the prevalence of multidrug-resistant organisms [10]. Moreover, vaccination and inappropriate antibiotic administration cause challenges in identifying patients with pneumococcal infections at an early stage. Previous studies have evaluated the clinical characteristics of paediatric patients with IPD and revealed several predictive variables associated with mortality, such as younger age, meningitis, and underlying diseases [11–14]. However, data on antimicrobial resistance and the risk factors for poor outcomes in children with IPD in China are limited.

In this study, we aimed to analyse the characteristics and antimicrobial resistance of pneumococcal strains and the risk factors for poor outcomes in paediatric patients with IPD in Hangzhou to identify a better strategy for reducing the incidence and mortality of IPD in China.

## Methods

### Study cohort

We performed a retrospective analysis using clinical data from the Pediatric Intensive Care (PIC) database [15], a large paediatric specific, single-center, bilingual database containing comprehensive clinical records between 2010 and 2018 from the Children’s Hospital of Zhejiang University School of Medicine, China [16]. A total of 178 paediatric patients with IPD and positive cultures of *S. pneumoniae* from the blood, CSF, pleural effusion, and other normally sterile body fluids were included in the study. Patients’ demographic details, vital signs, clinical examination results, and outcomes were documented. We analysed the first positive cultures obtained during the intensive care unit (ICU) stay of the enrolled patients. The Institutional Review Board of the Children’s Hospital of Zhejiang University School of Medicine (Hangzhou, China) reviewed and approved the study protocol. The requirement for individual patient consent was waived because of the retrospective observational nature of the study and the anonymisation of the medical records.

### Data collection

Clinical data, including demographic characteristics such as age and sex and vital signs such as temperature, heart rate (HR), respiratory rate (RR), systolic blood pressure (SBP), diastolic blood pressure (DBP), laboratory values, and outcomes, were collected. Infection and microorganism-associated variables included the sample type with a positive culture and antimicrobial resistance testing. Other laboratory values were obtained from the first blood sample collected after ICU admission. The primary diagnosis was based on the International Statistical Classification of Diseases and Related Health Problems, 10th edition (ICD-10) system. Sepsis was defined according to the Sepsis 3.0 as previously described [17].

### Antimicrobial resistance testing

Bacterial identification was performed using an automatic microorganism identification system (Vitek-2 Compact; bioMérieux, France). Antimicrobial resistance testing of *S. pneumoniae* was performed using the Vitek-2 AST-GP68 Test Kit according to the manufacturer’s instructions, as well as the E-test for penicillin resistance testing as supplementary experiments. The antibiotic susceptibility breakpoints for *S. pneumoniae* were determined at the time of detection in accordance with the latest edition of the guidelines of the Clinical and Laboratory Standards Institute at the time of detection.

### Statistical analysis

All the statistical analyses were performed using R version 3.4.3 [18, 19]. Continuous variables with a normal distribution were tested using the unpaired Student’s t-test and are presented as the mean ± standard deviation (SD), whereas non-normally distributed continuous variables were tested using the Mann–Whitney U test and are presented as the median (interquartile range, IQR). Categorical variables were tested using chi-square analysis or Fisher’s exact test and are presented as the frequencies (proportions). Univariate and multivariate logistic regression models, shown as odds ratios (ORs) and 95% confidence intervals (CIs), were used to identify factors associated with poor outcomes. Statistical significance was defined as a two-sided P-value of < 0.05.

## Results

### 1. Epidemiology and clinical characteristics

Over the 8 years from 2010 to 2018, 13,449 patients were admitted to the ICUs of the Children’s Hospital of Zhejiang University School of Medicine, and 178 patients (1.32%) had an IPD. Characteristics of the patients with and without IPDs are shown in Supplementary Table 1. Among the patients with IPD, 100 (56.18%) were male and 78 (43.82%) were female, with an average age of 22.63 (IQR 10.41–54.83) months. Among them, 2 (1.12%) patients were newborns (≤ 28 days), 51 (28.65%) patients were aged 29 days to 1 year, 118 (66.29%) patients were aged 2 to 10 years, and the remaining 7 (3.93%) patients were older than 11 years. A seasonal trend was observed, with 61.8% of all cases diagnosed during winter and spring, between December and May. The surgical intensive care unit (SICU) had the greatest number of patients with IPD (35.96%) while the cardiac intensive care unit (CICU) contained younger patients with IPD aged ≤ 1 year. The most frequent cause of ICU admission was cardiovascular disorder (30.34%). The respiratory tract (pleural effusion, 87.64%) was the most common site for positive *S. pneumoniae culture*, whereas newborns seemed more susceptible to bloodstream infections. Almost half of the patients with IPD (43.26%) used vasopressors during their ICU stay. The average length of ICU stay was 1.96 (IQR 0.91–6.14) days, and the average length of hospital stay was 13.85 (IQR 8.65–22.85) days. In total, 61 (34.27%) patients had sepsis, and seven patients died, with an in-hospital mortality rate of 3.93% (Table 1).

**Table 1:** Clinical and demographic characteristics of included patients in different age groups

	Total	Newborns (≤ 28 days)	Infancy (29 days to 1 years)	Childhood (2 years to 10 years)	Adolescence (11 years to 18 years)
Number	178	2	51	118	7
Age (months)	22.63 (10.41, 54.83)	0.40 (0.20, 0.60)	6.73 (3.87, 9.75)	38.27 (18.80, 63.93)	149.83 (146.56, 157.03)
Gender					
Male (%)	100 (56.18)	1 (50.00)	29 (56.86)	66 (55.93)	4 (57.14)
Female (%)	78 (43.82)	1 (50.00)	22 (43.14)	52 (44.07)	3 (42.86)
Disease onset season					
Dec-Feb (%)	68 (38.20)	0 (0.00)	24 (47.06)	41 (34.75)	3 (42.86)
Mar-May (%)	42 (23.60)	1 (50.00)	12 (23.53)	27 (22.88)	2 (28.57)
Jun-Aug (%)	34 (19.10)	1 (50.00)	8 (15.69)	24 (20.34)	1 (14.29)
Sep-Nov (%)	34 (19.10)	0 (0.00)	7 (13.73)	26 (22.03)	1 (14.29)
ICU type, n (%)					
PICU	43 (24.16)	0 (0.00)	10 (19.61)	32 (27.12)	1 (14.29)
CICU	53 (29.78)	1 (50.00)	18 (35.29)	33 (27.97)	1 (14.29)
NICU	2 (1.12)	1 (50.00)	1 (1.96)	0 (0.00)	0 (0.00)
SICU	64 (35.96)	0 (0.00)	16 (31.37)	44 (37.29)	4 (57.14)
General ICU	16 (8.99)	0 (0.00)	6 (11.76)	9 (7.63)	1 (14.29)
Primary diagnosis on ICU admission, n (%)					
Congenital	12 (6.74)	0 (0.00)	4 (7.84)	7 (5.93)	1 (14.29)
Hematological	5 (2.81)	0 (0.00)	0 (0.00)	5 (4.24)	0 (0.00)
Circulation	54 (30.34)	1 (50.00)	17 (33.33)	34 (28.81)	2 (28.57)
Neurologic	30 (16.85)	0 (0.00)	8 (15.69)	20 (16.95)	2 (28.57)
Digestive	10 (5.62)	0 (0.00)	3 (5.88)	5 (4.24)	2 (28.57)
Neoplasm	24 (13.48)	0 (0.00)	3 (5.88)	21 (17.80)	0 (0.00)
Respiratory	27 (15.17)	1 (50.00)	9 (17.65)	17 (14.41)	0 (0.00)
Trauma	6 (3.37)	0 (0.00)	1 (1.96)	5 (4.24)	0 (0.00)
Others	10 (5.62)	0 (0.00)	6 (11.76)	4 (3.39)	0 (0.00)
Sample with positive culture					
Blood	15 (8.43)	1 (50.00)	3 (5.88)	11 (9.32)	0 (0.00)
Cerebrospinal fluid	14 (7.87)	0 (0.00)	7 (13.73)	7 (5.93)	0 (0.00)
Pleural effusion	156 (87.64)	1 (50.00)	43 (84.31)	105 (88.98)	7 (100.00)
Vital signs					
Temperature (^o^C)	37.00 ± 0.74	35.60 ± 1.56	36.96 ± 0.55	37.04 ± 0.79	37.06 ± 0.37
HR (beats/min)	122.7 ± 20.12	133.00 ± 4.24	136.73 ± 16.03	116.12 ± 18.04	81.00 ± 0.00
RR (breaths/min)	30.26 ± 7.99	47.00 ± 9.90	35.79 ± 8.21	28.06 ± 6.31	22.48 ± 1.88
SBP (mmHg)	101.39 ± 15.47	52.50 ± 17.68	97.39 ± 15.11	103.52 ± 13.96	108.83 ± 12.32
DBP (mmHg)	58.89 ± 13.45	25.00 ± 7.07	55.95 ± 12.96	60.42 ± 12.96	64.33 ± 9.07
Vasopressor use during ICU stay time, n (%)	77 (43.26)	0 (0.00)	22 (43.14)	53 (44.92)	2 (28.57)
Hospital days	13.85 (8.65, 22.85)	34.53 (24.18, 44.87)	16.82 (9.11, 24.50)	12.04 (8.02, 20.99)	17.03 (11.28, 21.35)
ICU days	1.96 (0.91, 6.14)	27.73 (14.00, 41.47)	2.12 (0.92, 4.04)	1.91 (0.90, 7.05)	2.28 (1.68, 8.89)
Sepsis	61 (34.27)	1 (50.00)	21 (41.18)	36 (30.51)	3 (42.86)
In-hospital mortality	7 (3.93)	1 (50.00)	2 (3.92)	4 (3.39)	0 (0.00)

Abbreviations: Dec, December; Feb, February; Mar, March; Jun, June; Aug, August; Sep, September; Nov, November; ICU, intensive care unit; CICU, cardiac intensive care unit; NICU, neonatal intensive care unit; PICU, pediatric intensive care unit; SICU, surgical intensive care unit; HR, heart rate; RR, respiratory rate; SBP, systolic pressure; DBP, diastolic pressure

### 2. Antibiotic resistance

As shown in Table 2, antibiotic sensitivity tests were conducted with isolates from 163 patients (91.57%). The resistance (resistant + intermediate, R + I) of the *S. pneumoniae* isolates were highest for erythromycin (98.62%, 143 of 145), followed by tetracycline (90.06%, 145 of 161), and SMZ-Co (84.57%, 137 of 162). Penicillin resistance occurred in eight (72.73%) isolates from patients with meningitis, whereas penicillin resistance occurred in only six (4.05%) isolates from patients without meningitis. In addition, the resistance rates to ceftriaxone and cefotaxime were 45.45% and 54.55%, respectively, among isolates from patients with meningitis and 22.30% and 25.00%, among isolates from patients without meningitis. All isolates were sensitive to vancomycin, linezolid, moxifloxacin, telithromycin, ofloxacin, and levofloxacin. The number of multidrug-resistant (MDR) isolates (those resistant to ≥ three antibiotics at the same time) was 138 (84.66%).

**Table 2:** Antibiotic resistance results of pneumococcal isolates from children with invasive pneumococcal disease

	Total	No. of isolates (%)			
		S	I	*R*	*R* + I
Penicillin					
Meningitis	11	3 (27.27)	0 (0)	8 (72.73)	8 (72.73)
Nonmeningitis	148	142 (95.95)	2 (1.35)	4 (2.70)	6 (4.05)
Ceftriaxone					
Meningitis	11	6 (54.55)	3 (27.27)	2 (18.18)	5 (45.45)
Nonmeningitis	148	115 (77.70)	7 (4.73)	26 (17.57)	33 (22.30)
Cefotaxime					
Meningitis	11	5 (45.45)	5 (45.45)	1 (9.09)	6 (54.55)
Nonmeningitis	148	111 (75.00)	10 (6.76)	27 (18.24)	37 (25.00)
Erythromycin	145	2 (1.38)	0 (0)	143 (98.62)	143 (98.62)
Tetracycline	161	16 (9.94)	3 (1.86)	142 (88.20)	145 (90.06)
SMZ-Co	162	25 (15.43)	27 (16.67)	110 (67.90)	137 (84.57)
Chloramphenicol	160	154 (96.25)	0 (0)	6 (3.75)	6 (3.75)
Amoxicillin	106	88 (83.02)	6 (5.66)	12 (11.32)	18 (16.98)
Ertapenem	160	157 (98.13)	3 (1.88)	0 (0)	3 (1.88)
Vancomycin	163	163 (100)	0 (0)	0 (0)	0 (0)
Linezolid	160	160 (100)	0 (0)	0 (0)	0 (0)
Meropenem	159	58 (36.48)	87 (54.72)	14 (8.81)	101 (63.52)
Moxifloxacin	160	160 (100)	0 (0)	0 (0)	0 (0)
Telithromycin	160	160 (100)	0 (0)	0 (0)	0 (0)
Ofloxacin	158	158 (100)	0 (0)	0 (0)	0 (0)
Levofloxacin	162	162 (100)	0 (0)	0 (0)	0 (0)

Abbreviations: SMZ-Co, compound sulfamethoxazole; S, susceptible; I, intermediate; R, resistant

### 3. Comparison of clinical characteristics between survivors and nonsurvivors

Among the seven patients with IPD who died, six (85.71%) were male, with an average age of 17.97 (IQR 11.23–36.18) months (Table 3). The PICU and general ICU had the most nonsurvivors, whereas the SICU had the most survivors. Neurological diseases were observed in most patients (*n* = 3; 42.68%). A comparison of vital signs revealed that the differences in temperature, HR, RR, SBP, and DBP between the surviving and nonsurviving patients were not significant (all *P* > 0.05). A comparison of laboratory test results revealed that the PaO_2_ level (*P* = 0.030) was significantly greater in patients who survived than in those who died, whereas the D-dimer (*P* = 0.038), fibrinogen (Fib; *P* < 0.001), and C-reactive protein (CRP) levels (*P* = 0.002) were higher in patients who died than in those who survived. Among the patients with IPD who died, 28.57% used vasopressors, which was not significantly different from the percentage of surviving patients. In addition, the length of hospital stay was 4.41 (IQR 1.08–9.95) days in patients who died was significantly shorter than that in surviving patients (*P* = 0.045).

**Table 3:** Comparison of clinical characteristics between survivors and nonsurvivors of IPD children

	Survivors (*n* = 171)	Non-survivors (*n* = 7)	*P* value
Age (month)	23.40 (10.34, 55.47)	17.97 (11.23, 36.18)	0.582
Gender			0.108
Male (%)	94 (54.97)	6 (85.71)	
Female (%)	77 (45.03)	1 (14.29)	
ICU type, n (%)			0.009
PICU	40 (23.39)	3 (42.86)	
CICU	52 (30.41)	1 (14.29)	
NICU	2 (1.17)	0 (0.00)	
SICU	64 (37.43)	0 (0.00)	
General ICU	13 (7.60)	3 (42.86)	
Primary diagnosis on ICU admission, n (%)			0.327
Congenital	12 (7.02)	0 (0.00)	
Hematological	5 (2.92)	0 (0.00)	
Circulation	53 (30.99)	1 (14.29)	
Neurologic	27 (15.79)	3 (42.86)	
Digestive	10 (5.85)	0 (0.00)	
Neoplasm	24 (14.04)	0 (0.00)	
Respiratory	26 (15.20)	1 (14.29)	
Trauma	5 (2.92)	1 (14.29)	
Others	9 (5.26)	1 (14.29)	
Vital signs			
Temperature, ^o^C	36.98 ± 0.71	38.02 ± 1.28	0.116
HR (beats/min)	122.59 ± 20.12	125.67 ± 24.21	0.797
RR (breaths/min)	29.92 ± 7.56	40.34 ± 13.98	0.114
SBP (mmHg)	101.35 ± 15.07	102.60 ± 27.78	0.860
DBP (mmHg)	58.85 ± 13.09	60.40 ± 24.39	0.800
WBC (×10^9^ /L)	9.78 (7.17, 13.28)	12.04 (9.34, 15.09)	0.781
PLT (×10^9^ /L)	314.00 (236.50, 408.00)	279.00 (252.00, 316.50)	0.583
Hemoglobin (g/L)	118.00 (104.50, 127.00)	115.00 (101.50, 129.25)	0.879
LDH (U/L)	320.00 (260.00, 408.00)	583.50 (328.25, 670.00)	0.312
PH	7.39 ± 0.08	7.32 ± 0.17	0.057
PaCO_2_ (mmHg)	36.30 (32.45, 40.00)	37.80 (29.75, 52.90)	0.719
PaO_2_ (mmHg)	137.00 (76.35, 179.50)	66.10 (47.22, 110.92)	0.030
ALT (U/L)	17.00 (12.00, 29.00)	13.50 (10.25, 29.50)	0.992
CK-MB (U/L)	31.00 (23.00, 45.00)	25.00 (22.50, 60.50)	0.525
Creatinine (umol/L)	43.00 (37.00, 49.00)	52.50 (41.00, 55.75)	0.644
Sodium (mmol/L)	136.19 ± 4.37	137.17 ± 6.31	0.598
INR	0.98 (0.93, 1.07)	1.06 (0.99, 1.14)	0.743
APTT (s)	30.30 (27.10, 34.80)	31.05 (28.18, 36.78)	0.904
PT (s)	11.80 (11.10, 12.80)	12.75 (11.93, 13.80)	0.731
D-dimer (mg/L)	0.71 (0.24, 2.35)	6.43 (4.66, 7.34)	0.038
Fib (g/L)	2.30 (1.87, 3.07)	4.55 (3.29, 5.97)	< 0.001
CRP (mg/L)	7.12 (4.00, 34.25)	72.00 (39.00, 164.00)	0.002
Lactate (mmol/L)	1.50 (0.90, 2.50)	1.90 (1.15, 4.30)	0.268
Albumin (g/L)	42.10 (37.05, 44.70)	40.65 (33.62, 42.50)	0.597
Vasopressor use during ICU stay time, n (%)	75 (43.86)	2 (28.57)	0.141
Hospital days	14.06 (8.92, 22.94)	4.41 (1.08, 9.95)	0.045
ICU days	1.96 (0.91, 6.09)	1.13 (0.85, 6.26)	0.525

Abbreviations: ICU, intensive care unit; CICU, cardiac intensive care unit; NICU, neonatal intensive care unit; PICU, pediatric intensive care unit; SICU, surgical intensive care unit; HR, heart rate; RR, respiratory rate; SBP, systolic pressure; DBP, diastolic pressure; WBC, white blood cell; PLT, platelet; LDH, lactic dehydrogenase; PaCO_2,_ arterial partial pressure of carbon dioxide; PaO_2_, arterial oxygen partial pressure; ALT, alanine transaminase; CK-MB, creatine kinase-MB; INR, international normalized ratio; APTT, activated partial thromboplastin time; PT, prothrombin time; Fib, fibrinogen; CRP, C reactive protein

### 4. Risk factors for sepsis among patients with IPD

Among the 61 patients with IPD who developed sepsis, 35 (57.38%) were male, with an average age of 18.40 (IQR 6.87–55.97) months (Table 4). Among the patients with IPD, the PICU had the most patients with sepsis (45.90%), whereas the SICU had the most patients without sepsis (42.74%). In patients with sepsis, the most frequent causes of ICU admission were neurological and respiratory disorders (both 24.59%). Higher temperatures, HR, and RR were found in patients with sepsis than in those without (all *P* < 0.05). A comparison of laboratory test results revealed that the international normalised ratio (INR), prothrombin time (PT), D-dimer levels, CRP levels, and lactate levels were significantly higher in patients with sepsis than in those without sepsis. In paediatric patients with IPD who developed sepsis, the platelet, haemoglobin, PaO_2_, and albumin levels were significantly lower than those in patients without sepsis. Hospital data showed that the duration of hospital and ICU stays in the sepsis group were significantly longer than those in the nonsepsis group (both *P* < 0.001). The difference in the in-hospital mortality rate between the sepsis group and nonsepsis groups was not statistically significant (Table 4). Temperature (OR 3.80, 95% CI 1.62–8.87; *P* = 0.0021), PaO_2_ (OR 0.99, 95% CI 0.98-1.00; *P* = 0.0266), and albumin (OR 0.89, 95% CI 0.80–0.99; *P* = 0.0329) were found to be independent risk factors for sepsis in children with IPD (Table 5).

**Table 4:** Clinical characteristics of invasive pneumococcal disease in children with or without sepsis

	No sepsis (*n* = 117)	Sepsis (*n* = 61)	*P* value
Age (month)	24.43 (11.77, 54.40)	18.40 (6.87, 55.97)	0.863
Gender			0.816
Male (%)	65 (55.56)	35 (57.38)	
Female (%)	52 (44.44)	26 (42.62)	
ICU type, n (%)			< 0.001
PICU	15 (12.82)	28 (45.90)	
CICU	44 (37.61)	9 (14.75)	
NICU	1 (0.85)	1 (1.64)	
SICU	50 (42.74)	14 (22.95)	
General ICU	7 (5.98)	9 (14.75)	
Primary diagnosis on ICU admission, n (%)			0.001
Congenital	9 (7.69)	3 (4.92)	
Hematological	1 (0.85)	4 (6.56)	
Circulation	46 (39.32)	8 (13.11)	
Neurologic	15 (12.82)	15 (24.59)	
Digestive	7 (5.98)	3 (4.92)	
Neoplasm	17 (14.53)	7 (11.48)	
Respiratory	12 (10.26)	15 (24.59)	
Trauma	5 (4.27)	1 (1.64)	
Others	5 (4.27)	5 (8.20)	
Vital signs			
Temperature, ^o^C	36.82 ± 0.52	37.42 ± 0.96	< 0.001
HR (beats/min)	118.37 ± 18.61	133.10 ± 20.17	0.003
RR (breaths/min)	28.27 ± 6.39	34.49 ± 9.36	< 0.001
SBP (mmHg)	101.17 ± 15.11	101.87 ± 16.34	0.790
DBP (mmHg)	58.02 ± 13.58	60.71 ± 13.13	0.237
WBC (×10^9^ /L)	9.75 (7.23, 13.11)	9.89 (6.97, 14.09)	0.186
PLT (×10^9^ /L)	323.00 (254.00, 404.00)	280.50 (188.75, 410.00)	0.018
Hemoglobin (g/L)	120.00 (109.00, 128.00)	111.50 (98.00, 124.25)	0.007
LDH (U/L)	306.00 (260.50, 386.00)	346.00 (258.50, 576.00)	0.112
PH	7.39 ± 0.09	7.38 ± 0.08	0.687
PaCO2 (mmHg)	35.80 (32.90, 40.00)	36.60 (32.00, 41.60)	0.736
PaO2 (mmHg)	154.00 (98.60, 190.00)	96.50 (64.67, 128.75)	< 0.001
ALT (U/L)	16.00 (12.00, 26.00)	21.50 (12.00, 45.50)	0.061
CK-MB (U/L)	32.00 (24.00, 44.50)	30.00 (21.00, 45.50)	0.417
Creatinine (umol/L)	43.00 (38.00, 48.00)	43.00 (35.60, 52.25)	0.781
Sodium (mmol/L)	136.45 ± 3.82	135.78 ± 5.43	0.342
INR	0.97 (0.93, 1.04)	1.03 (0.94, 1.17)	0.002
APTT (s)	30.20 (27.00, 34.60)	30.90 (27.20, 36.18)	0.341
PT (s)	11.70 (11.10, 12.50)	12.30 (11.30, 14.05)	0.002
D-dimer (mg/L)	0.52 (0.19, 1.82)	1.42 (0.48, 3.47)	0.021
Fib (g/L)	2.27 (1.90, 2.86)	2.44 (1.72, 3.70)	0.215
CRP (mg/L)	6.00 (3.00, 30.17)	14.38 (5.25, 72.25)	0.003
Lactate (mmol/L)	1.30 (0.80, 2.30)	1.90 (1.30, 2.60)	0.003
Albumin (g/L)	43.00 (40.30, 45.50)	37.55 (31.60, 42.85)	< 0.001
Vasopressor use during ICU stay time, n (%)	55 (47.01)	22 (36.07)	0.292
Hospital days	10.94 (7.17, 14.99)	23.61 (16.82, 35.72)	< 0.001
ICU days	1.13 (0.84, 3.01)	7.08 (2.28, 22.59)	< 0.001
In-hospital mortality	5 (4.27%)	2 (3.28%)	0.746

Abbreviations: ICU, intensive care unit; CICU, cardiac intensive care unit; NICU, neonatal intensive care unit; PICU, pediatric intensive care unit; SICU, surgical intensive care unit; HR, heart rate; RR, respiratory rate; SBP, systolic pressure; DBP, diastolic pressure; WBC, white blood cell; PLT, platelet; LDH, lactic dehydrogenase; PaCO_2,_ arterial partial pressure of carbon dioxide; PaO_2_, arterial oxygen partial pressure; ALT, alanine transaminase; CK-MB, creatine kinase-MB; INR, international normalized ratio; APTT, activated partial thromboplastin time; PT, prothrombin time; Fib, fibrinogen; CRP, C reactive protein

**Table 5:** Logistic regression analysis for risk factors of sepsis in IPD children

	Univariate analysis		Multivariable analysis	
	OR (95% CI)	*P* value	OR (95% CI)	*P* value
Temperature, ^o^C	3.57 (1.98, 6.46)	< 0.0001	3.80 (1.62, 8.87)	0.0021
PLT (×10^9^ /L)	1.00 (0.99, 1.00)	0.0200	1.00 (1.00, 1.00)	0.8748
Hemoglobin (g/L)	0.98 (0.96, 0.99)	0.0092	1.00 (0.97, 1.04)	0.7767
PaO2 (mmHg)	0.99 (0.98, 0.99)	< 0.0001	0.99 (0.98, 1.00)	0.0266
INR	8.77 (1.83, 41.99)	0.0066	3.03 (0.39, 23.61)	0.2890
D-dimer (mg/L)	1.12 (1.01, 1.25)	0.0333	0.95 (0.80, 1.13)	0.5622
CRP (mg/L)	1.01 (1.00, 1.02)	0.0055	1.00 (0.99, 1.01)	0.6369
Lactate (mmol/L)	1.16 (0.94, 1.44)	0.1681		
Albumin (g/L)	0.87 (0.82, 0.92)	< 0.0001	0.89 (0.80, 0.99)	0.0329

Abbreviations: PLT, platelet; PaO_2_, arterial oxygen partial pressure; INR, international normalized ratio; CRP, C reactive protein

## Discussion

IPD is a significant health concern in children worldwide. We conducted a retrospective single-center study involving patients with IPD in the ICUs of a specialised hospital in Hangzhou, China, from 2010 to 2018. This study focused on the epidemiology, bacterial antimicrobial resistance, and prognostic prediction of patients with IPD with the aim of enhancing our understanding and early treatment of this disease in paediatric patients admitted to the ICU.

The epidemiology of IPD in children varies according to region and age. In our cohort, 66.29% of children with IPD were aged 2–10 years, which differs from data from other studies [3, 20]. Among patients with IPD, the overall mortality rate was 3.93%, which was lower than that reported in other studies, where the mortality rate was 6.25-7.8% [21]. More than half (61.8%) of patients developed IPD during the cold season. Understanding the epidemiology of IPD will aid in the development of effective preventive measures. Various underlying diseases, including cancer, asthma [22], renal disease, congenital heart disease (CHD), and immune deficiency [23] are often observed in patients with IPD and are considered risk factors for IPD. In our study, cardiovascular disorders, especially CHD, were the most common underlying diseases in children with IPD, indicating strong susceptibility to *S. pneumoniae* infection in these patients.

Antimicrobial resistance is a growing challenge in the treatment of IPD in children worldwide, particularly in Asia. Data from the Asian Network for Surveillance of Resistant Pathogens showed that China has the highest rate of multidrug and macrolide resistance among *S. pneumoniae* isolates [24]. In the present study, the rate of erythromycin resistance was the highest in *S. pneumoniae*, which is similar to findings in other regions of China, including Suzhou [20], Shanghai [25], and Beijing [26]. However, the proportion of erythromycin-resistant isolates in India is approximately 37% [27], which is lower than that in China. Consistent with the previous findings [3], our study revealed high rates of resistance to tetracycline and SMZ-Co. Notably, the resistance rates to penicillin, ceftriaxone, and cefotaxime were significantly higher in the meningeal isolates than in the non-meningeal isolates. Therefore, different infectious conditions should be considered during individual antimicrobial selection in patients with and without meningitis. Moreover, all isolates were sensitive to vancomycin, linezolid, moxifloxacin, telithromycin, ofloxacin, and levofloxacin. Another study of 123 hospitalised children with IPD in Shanghai, China, also showed that vancomycin, linezolid, and levofloxacin were sensitive antibiotics for all detected *S. pneumoniae* isolates [3]. In our study, the proportion of observed MDR bacteria (84.66%) was similar to that reported in another study in China (81.2%) [28] and other Asian countries (83.3%) [24] but was substantially greater than that in India [27], reflecting the high geographical and genetic diversity of *S. pneumoniae*. Appropriate surveillance and antibiotic stewardship are crucial for the management of resistant strains.

Patients with IPD may experience poor outcomes including death and sepsis. In this study, the in-hospital mortality rate of children with IPD was 3.93%, and most nonsurvivors were males aged < 5 years. In addition, 61 (34.27%) patients with IPD developed sepsis and had longer hospital and ICU stays, indicating severe infection complicated by organ dysfunction. Predicting risk factors for poor prognosis in children with IPD aids in the early identification of severe cases and guides treatment decisions. Previous studies have shown that age, meningitis, underlying diseases, penicillin resistance, and inappropriate initial antibiotic therapy are risk factors for IPD-related mortality [11, 29, 30]. Our results showed that PaO_2_ levels decreased significantly in nonsurvivors and were independent predictors of sepsis in children with IPD. Similarly, a retrospective single-centre study in Beijing indicated that respiratory failure was an independent risk factor for mortality in patients with IPD [28]. Therefore, preventing hypoxemia and ensuring a sufficient oxygen supply are effective measures for reducing the mortality of patients with IPD.

Dysfunction of coagulation plays a crucial role in the pathogenesis of sepsis-related host dysregulation and organ failure. Increasing evidence suggests that the D-dimer is a valuable biomarker for predicting the prognosis of patients with different infectious conditions [31–34]. In the present study, D-dimer levels were significantly higher in patients who died and in those with sepsis. A D-dimer cutoff value of 3,000 ng/mL helps in identifying patients with a two-fold increase in the occurrence of multiple organ dysfunction syndrome in *S. pneumoniae-*invasive infections [35]. In addition, Fib levels were higher in patients who died than in survivors, and the INR and PT were significantly higher in patients with sepsis. These results highlight that medical staff should especially note patients with IPD and coagulopathy and ensure prompt management to reduce the risk of poor outcomes.

As an indicator of inflammation, CRP levels were significantly higher in nonsurvivors and patients with sepsis in our study. A previous study that compared IPD and pneumococcal pneumonia showed that a CRP level ≥ 17.0 mg/dL was a useful factor for suspicion of IPD [36]. Similarly, high CRP levels, leukopenia and thrombocytopenia are associated with mortality in paediatric patients with IPD, and these factors warrant special attention upon admission [37]. Moreover, we found that temperature (OR 3.80, 95% CI 1.62–8.87) was an independent predictor of sepsis in children with IPD, indicating that severe inflammation participated in disease progression. Intense inflammation is a hallmark of IPD and appropriate anti-inflammatory treatment is necessary.

Serum albumin (ALB) levels are also an independent risk factor for sepsis in children with IPD. Hypoalbuminaemia is common in critically ill patients because of decreased hepatocyte synthesis and altered distribution from vessels to tissues [38]. Recently, increasing evidence has indicated that decreased serum ALB concentration is a marker of inflammation rather than that of nutritional status [39, 40]. A nationwide multicentre study of 3967 patients with sepsis admitted to the ICU revealed that a decrease in serum ALB concentration one week after admission was a strong predictor of mortality in younger adults [41]. Additionally, low serum ALB levels are associated with an increased risk of IPD [42]. ALB-containing solutions are recommended for the resuscitation of patients with sepsis because of the lower mortality rate of patients treated with these solutions than that of patients treated with other fluid resuscitation strategies [43]. However, another multicentre study showed that compared with crystalloids alone, ALB replacement in combination with crystalloids did not improve the survival rate at 28 or 90 days in patients with severe sepsis [44]. Therefore, additional high-quality studies are required to explore the effects of ALB replacement therapy.

This study has several limitations. First, owing to its retrospective, single-centre nature, the sample size was relatively small. However, as one of the national regional medical centres for children, a substantial proportion of inpatients are from all over the country; thus, they could, at least in part, represent the clinical characteristics of IPD. Second, the serotypes of *S. pneumoniae* isolates were not determined. Third, our study did not include paediatric patients with non-IPDs in the ICUs, which may create a bias. In addition, because of the lack of data concerning vaccination of patients in the PIC database, we did not show relevant data on immunisation with PCVs. Therefore, large-scale multicentre studies, including the serotyping of pneumococcal isolates and immunisation with PCVs, are required in the future.

## Conclusions

This study demonstrated that paediatric IPD deserves attention in China. Most children with PID in the ICUs were 2–10 years old. Antimicrobial resistance tests of *S. pneumoniae* isolates revealed high resistance to erythromycin, tetracycline, and SMZ-Co. All isolates were sensitive to vancomycin, linezolid, moxifloxacin, telithromycin, ofloxacin, and levofloxacin. Patients with IPD may experience poor outcomes including death and sepsis. The in-hospital mortality rate was 3.93%, and 34.27% of patients had sepsis. Temperature, PaO_2_ levels, and serum ALB concentration were independent risk factors for sepsis in children with IPD. Considering that this was a retrospective single-centre study, further prospective, multicentre studies are required to validate our findings.

### Electronic supplementary material

Below is the link to the electronic supplementary material.

Supplementary Material 1

## Data Availability

The datasets used and/or analysed during the current study are available from the corresponding author on reasonable request.

## Abbreviations

IPD: Invasive pneumococcal disease
PIC: Pediatric intensive care
ICUs: Intensive care units
SMZ-Co: Compound sulfamethoxazole
S. pneumoniae: Streptococcus pneumoniae
CSF: Cerebrospinal fluid
PCVs: Pneumococcal conjugate vaccines
MDROs: Multidrug-resistant organisms
HR: Heart rate
RR: Respiratory rate
SBP: Systolic blood pressure
DBP: Diastolic blood pressure
SD: Standard deviation
IQR: Interquartile range
ORs: Odds ratios
CIs: Confidence intervals
SICU: Surgical intensive care unit
CICU: Cardiac intensive care unit
MDR: Multidrug-resistant
PaO_2_: Partial Pressure of Oxygen in Arterial Blood
Fib: Fibrinogen
CRP: C-reactive protein
INR: International normalized ratio
PT: Prothrombin time
CHD: Congenital heart disease
MODS: Multiple organ dysfunction syndrome
ALB: Albumin
