# Supplementary material for: Clinical characteristics and risk factors for poor outcomes of invasive pneumococcal disease in pediatric patients in China
Source: BMC Infect Dis. 2024 Jun 19;24:602. doi: 10.1186/s12879-024-09493-9 (PMC11186143; doi:10.1186/s12879-024-09493-9)
Supplement: Supplementary file 1 — Supplementary Material 1 [file 12879_2024_9493_MOESM1_ESM.docx]

Supplementary table 1 Clinical and demographic characteristics of patients with IPD and without IPD.

|  | Total  (n = 13449) | Non-IPD patients  (n = 13271) | IPD patients  (n = 178) | *P* value |
| --- | --- | --- | --- | --- |
| Age (months) | 8.77 (1.30, 41.57) | 8.60 (1.23, 41.10) | 22.63 (10.41, 54.83) | 0.038 |
| Male (%) | 7728 (57.46) | 7628 (57.48) | 100 (56.18) | 0.728 |
| ICU type, n (%) |  |  |  | < 0.001 |
| PICU | 2081 (15.47) | 2038 (15.36) | 43 (24.16) |  |
| CICU | 2655 (19.74) | 2602 (19.61) | 53 (29.78) |  |
| NICU | 3196 (23.76) | 3194 (24.07) | 2 (1.12) |  |
| SICU | 2792 (20.76) | 2728 (20.56) | 64 (35.96) |  |
| General ICU | 2725 (20.26) | 2709 (20.41) | 16 (8.99) |  |
| Primary diagnosis on ICU admission, n (%) |  |  |  | < 0.001 |
| Congenital | 2684 (19.96) | 2672 (20.13) | 12 (6.74) |  |
| Hematological | 464 (3.45) | 459 (3.46) | 5 (2.81) |  |
| Circulation | 2664 (19.81) | 2610 (19.67) | 54 (30.34) |  |
| Neurologic | 1379 (10.25) | 1349 (10.17) | 30 (16.85) |  |
| Digestive | 1235 (9.18) | 1225 (9.23) | 10 (5.62) |  |
| Neoplasm | 979 (7.28) | 955 (7.20) | 24 (13.48) |  |
| Respiratory | 1986 (14.77) | 1959 (14.76) | 27 (15.17) |  |
| Trauma | 582 (4.33) | 576 (4.34) | 6 (3.37) |  |
| Others | 1476 (10.98) | 1466 (11.05) | 10 (5.62) |  |
| Vasopressor use during ICU  stay time, n (%) | 4107 (30.54) | 4030 (30.37) | 77 (43.26) | < 0.001 |
| Hospital days | 12.34 (7.03, 20.97) | 12.26 (7.02, 20.96) | 13.85 (8.65, 22.85) | 0.960 |
| ICU days | 2.84 (0.93, 9.75) | 2.85 (0.93, 9.78) | 1.96 (0.91, 6.14) | 0.059 |
| In-hospital mortality | 971 (7.22) | 964 (7.26) | 7 (3.93) | 0.088 |

Abbreviations: ICU, intensive care unit; CICU, cardiac intensive care unit; NICU, neonatal intensive care unit; PICU, pediatric intensive care unit; SICU, surgical intensive care unit.
